# A Comparative Study between Blended Polymers and Copolymers as Emitting Layers for Single-Layer White Organic Light-Emitting Diodes

**DOI:** 10.3390/ma17010076

**Published:** 2023-12-23

**Authors:** Despoina Tselekidou, Kyparisis Papadopoulos, Vasileios Foris, Vasileios Kyriazopoulos, Konstantinos C. Andrikopoulos, Aikaterini K. Andreopoulou, Joannis K. Kallitsis, Argiris Laskarakis, Stergios Logothetidis, Maria Gioti

**Affiliations:** 1 Nanotechnology Lab LTFN, Department of Physics, Aristotle University of Thessaloniki, GR-54124 Thessaloniki, Greece; kypapado@physics.auth.gr (K.P.); alask@physics.auth.gr (A.L.); logot@auth.gr (S.L.); 2 Organic Electronic Technologies P.C. (OET), 20th KM Thessaloniki—Tagarades, GR-57001 Thermi, Greece; v.kyriazopoulos@oe-technologies.com; 3 Department of Chemistry, University of Patras, Caratheodory 1, University Campus, GR-26504 Patras, Greece; k.andrikopoulos@ac.upatras.gr (K.C.A.); andreopo@upatras.gr (A.K.A.); j.kallitsis@upatras.gr (J.K.K.)

**Keywords:** WOLEDs, single-layer, blend polymers, copolymers, energy transfer

## Abstract

Extensive research has been dedicated to the solution-processable white organic light-emitting diodes (WOLEDs), which can potentially influence future solid-state lighting and full-color flat-panel displays. The proposed strategy based on WOLEDs involves blending two or more emitting polymers or copolymerizing two or more emitting chromophores with different doping concentrations to produce white light emission from a single layer. Toward this direction, the development of blends was conducted using commercial blue poly(9,9-di-n-octylfluorenyl2,7-diyl) (PFO), green poly(9,9-dioctylfluorenealt-benzothiadiazole) (F8BT), and red spiro-copolymer (SPR) light-emitting materials, whereas the synthesized copolymers were based on different chromophores, namely distyryllanthracene, distyrylcarbazole, and distyrylbenzothiadiazole, as yellow, blue, and orange–red emitters, respectively. A comparative study between the two approaches was carried out to examine the main challenge for these doping systems, which is ensuring the proper balance of emissions from all the units to span the entire visible range. The emission characteristics of fabricated WOLEDs will be explored in terms of controlling the emission from each emitter, which depends on two possible mechanisms: energy transfer and carrier trapping. The aim of this work is to achieve pure white emission through the color mixing from different emitters based on different doping concentrations, as well as color stability during the device operation. According to these aspects, the WOLED devices based on the copolymers of two chromophores exhibit the most encouraging results regarding white color emission coordinates (0.28, 0.31) with a CRI value of 82.

## 1. Introduction

White organic light-emitting diodes (WOLEDs), produced through a solution deposition process, offer a formidable opportunity for low-cost, environmentally friendly, and flexible light sources capable of meeting the spectral, functional, and budgetary needs of emerging application fields. These fields include next-generation full-color displays and solid-state lighting [1,2,3,4,5,6,7]. Until now, the reported high-efficiency WOLEDs with multilayer device structures have been mostly fabricated through vacuum evaporation [8]. Although such complex device structures can achieve high efficiency, the complicated process and high economic cost are not suitable for further industrial application. In this context, a device processing procedure based on solutions, such as spin-coating, slot die coating, etc., provides numerous advantages compared to a vacuum deposition process. Aside from the low cost and mass production, solution-processed OLEDs hold an edge over other techniques, since they can also be designed in many different shapes and designs, opening an enormous array of design possibilities [9].

The most straightforward way to obtain white light is to integrate luminophores of different wavelengths in the same device or even in a single emitting layer [10,11,12]. Especially, the widely adopted methods for fabricating white OLEDs are either stacking multiple emitting layers or mixing different colors in one emitting layer (EML). Among these strategies, multilayer polymer LED fabrication is more laborious, as their fabrication is more difficult and there is a risk that the solvent may destroy the underlayers. On the other hand, the alternative strategy based on single-emissive layer WOLEDs has shown to be a more reliable option in scientific and industrial communities because of the low fabrication costs, simple structure, and easy solution processing [4,13,14].

More specifically, the output spectral range of an OLED must span the entire visible range (400–800 nm) in order to be considered a preferred and optimal white light source. Therefore, a combination of two or three emissive materials is usually required for efficient and easy color tuning to achieve white EL emission, because only one emissive material cannot cover a sufficient spectral range. According to this demand, the white light emission could be generated by mixing three primary colors (blue, green, and red) or complementary colors (red or yellow and blue) to cover the entire visible region. There have been several proposed luminescent material systems, which have been devised resulting in the exciton regulation to emit white light in a balanced manner. Regarding this issue, two of the proposed strategies involve either the blend of two or more light-emitting polymers [15,16] or the use of multicolor-emitting copolymers bearing different chromophores [4,14]. It is worth noting that for producing environmentally friendly and practical WOLEDs, fluorescent materials are superior to phosphorescent materials, as the former do not contain any heavy metals [17]. In addition, compared to small molecules, the emitting polymers are deemed more suitable for low-cost and large-area wet methods [18].

More specifically, in the case of blended materials, some well-studied binary blends, based on blue and red emitters, are the combination of poly [9,9-dioctylfluorenyl-2,7-diyl] (PFO) with poly(2-methoxy-5(2-ethylhexyl)-1,4-phenylenevinylene (MEH-PPV) [19] or poly [2-methoxy-5-(3,7-dimethyl-octyloxy)-1,4-phenylenevinylene] capped with dimethylphenyl (MDMO-PPV-DMP) [20]. More specifically, Prakash et al. suggested, as an emissive layer, the binary blended PFO-MEH-PPV for WOLED and exhibiting Commission Internationale de l’Enclairage (CIE) coordinates of (0.30, 0.38) and luminance value reaching 1234 cd/m^2^ at 8.5 V [19]. The ternary blended materials are based on PFO, MEH-PPV, and incorporation into a green emitter, such as poly 9,9-dioctylfluorene-altbenzothiadiazole (F8BT) [21] or 2-butyl-6-(butylamino)benzo(de)isoquinoline-1,3-dione (F7GA) [22]. Al-Asbahi studied the latter case PFO-F7GA-MEH-PPV and obtained CIE coordinates of (0.31, 0.24) and the luminance value of 2295 cd/m^2^ [22]. For the synthesized materials, Kim et al. present, as an emissive layer, the copolymer, which consists of polyfluorene (PFO) and polytriarylamine (PTAA) with a benzothiadiazole (BZ) moiety and exhibiting CIE coordinates of (0.34, 0.41) and a luminance value of 320 cd/m^2^ [23]. However, for all studied cases, there is still a need to optimize the emission and operating characteristics of WOLEDs.

Both the scenarios of blended and copolymers as emitting layers mentioned above are founded on a host–guest system. An ideal host–guest system should emit a continuous spectrum of different colors spanning the entire visible range. The efficient control and tuning of emission colors are crucial. The diffusion of excitons is a vital factor in the operation of OLEDs, particularly WOLEDs, and their control is influenced by the doping concentration. More specifically, for the efficient WOLED, excitons need to be distributed to various emitters. It is well established that the white emission can be realized through the Förster resonance energy transfer (FRET) from a wide-band-gap host molecule to a low-band-gap dopant molecule. The requirements for efficient energy transfer are as follows: (i) the donor should possess a high photoluminescence quantum yield (PLQY), (ii) the acceptor species should be located in close proximity to the donor species, and most importantly, (iii) it should satisfy the fundamental criterion of excellent spectral overlap in between the emission of the donor and absorption of the acceptor [22,24,25]. Part of the energy is transferred to the guest emitter, allowing for color mixing with its self-emission. However, the efficient energy transfer from the host to the dopant material often requires precise control of the guest doping concentration, thus increasing the challenge of device preparation. Hence, the key challenge lies in discovering a host emission and dopants most fitting while considering the doping concentration, which is an equally critical aspect to attain emission in the whole visible range. Accomplishing pure white light emission that offers commendable CRI values and CIE coordinates proximate to the ideal white point of (0.33, 0.33) remains the ultimate objective.

With the aim of advancing solution-processable WOLEDs toward their full potential, herein, we investigate light-emitting blended polymers or multicolor copolymers, bearing different chromophores, as a route to achieve the emission in the whole visible range for lighting applications. In particular, commercial blue poly(9,9-di-n-octylfluorenyl2,7-diyl) (PFO), green poly(9,9-dioctylfluorenealt-benzothiadiazole) (F8BT), and red spiro-copolymer (SPR) light-emitting materials are used to develop blends, and distyrylanthracene, distyrylcarbazole, and distyrylbenzothiadiazole chromophores as yellow, blue, and orange–red emitters, respectively, are used to synthesize novel copolymers. Since the host and dopant molecules emit at different visible-spectrum wavelengths, adjusting the concentration ratio could facilitate white light emission. The proper tuning of this ratio holds significant potential for generating white light. The optical and photophysical properties of the emitting films are thoroughly investigated through Spectroscopic Ellipsometry and Photoluminescence. Following this, these materials are applied as emitting layers in solution-processable single-layer OLEDS, via the spin coating method, and the device architecture is the simplest and most commonly used. We assess the WOLEDs produced based on their electroluminescence properties, including their luminance, CIE coordinates, CRI, and CCT values. This study aims to compare various emitting systems with different doping concentrations to investigate the photo- and electroluminescence characteristics. Subsequently, the most suitable emitting system will be evaluated based on its most promising results toward the attainment of pure white light emission.

## 2. Materials and Methods

### 2.1. Ink Formulation

Poly-3,4-ethylene dioxythiophene:poly-styrene sulfonate (PEDOT:PSS, purchased from Clevios Heraus, Hanau, Germany) AI 4083, mixed with ethanol in the ratio of 2:1, was prepared and used as the hole transport layer (HTL) in produced OLEDs. For the blend emitting polymers, the blue emitting poly(9,9-di-n-octylfluorenyl-2,7-diyl) PFO (Mw = 114,050) and the green emitting poly(9,9-dioctylfluorene-alt-benzothiadiazole) F8BT (Mw = 237,460) were purchased from Ossila (Sheffield, UK), and the red emitting spiro-copolymer-001 SPR (Mw = 720,000) was purchased from Sigma-Aldrich Chemie GmbH, Taufkirchen, Germany. All materials were dissolved in a toluene solvent. SPR and PFO solutions with different weight ratios, stirred under heating at 50 °C for 24 h, were used to prepare the homogeneous binary blends. In the same manner, SPR, F8BT, and PFO solutions were used to prepare the ternary blends. Analytically, the ratios used are listed in Table 1, where the codes of studied samples and the blend type are included. For all solutions, the final concentration was fixed at 1% *w*/*w*.

**Scheme 1:**
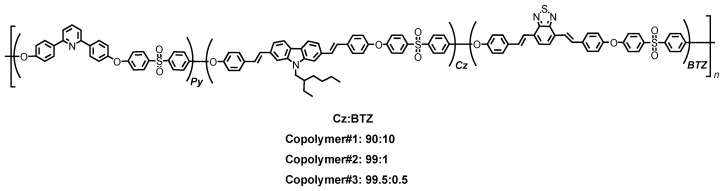
Chemical structure of copolymers bearing two emissive chromophores.

**Scheme 2:**
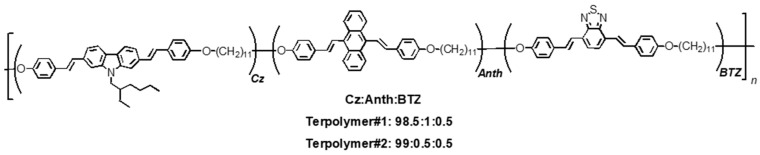
Chemical structure of terpolymers bearing three emissive chromophores.

In the case of the synthesized copolymers Cz:BTZ with ratios 90:10, 99:1, and 99.5:0.5, the molecular weights of the copolymers were estimated at Mn = 49,800, 45,100, and 34,900, respectively. More details on the synthetic processes are given in refs. [4,14,26]. The molecular weights for terpolymers with ratios 98.5:1.0:0.5 and 99:0.5:0.5 were estimated at 9540 and 8800. The chemical structures of copolymers and terpolymers are shown in Scheme 1 and Scheme 2, respectively. The copolymers were dissolved in N, N-dimethylformamide, DMF, with a resulting concentration of 1% wt, and the terpolymers were dissolved in o-DCB:CHCl_3_ (9:1) with a resulting concentration of 1% wt [4,26]. The ratios for the copolymers and terpolymers are summarized in Table 2, where the codes of studied samples and the copolymer type are included.

### 2.2. OLED Fabrication

**Scheme 3:**
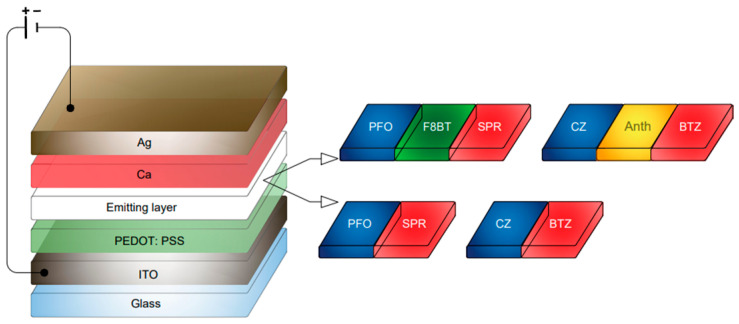
The architecture of fabricated OLED devices with either blends or copolymers forming the photoactive layers.

The solution-processed WOLED structure is shown in Scheme 3. Initially, the glass substrates (provided by Ossila, Sheffield, UK) precoated with indium tin oxide (ITO) layers were cleaned in an ultrasonic bath by sequentially immersing them in deionized water, acetone, and ethanol for 10 min each. The substrates were then dried with ultrapure nitrogen gas and subsequently underwent oxygen plasma treatment at 40 W for 3 min. Afterward, the PEDOT:PSS ink was filtered (using PTFE filters with a 0.45 µm pore size) and spin-coated on the substrates at 4500 rpm for 60 s. The samples were then annealed on a hot plate glass/ITO/PEDOT:PSS at 120 °C for 5 min. The photoactive layer solution was then spin-coated at 2000 rpm for 25 s onto the samples without further annealing. Finally, by using a shadow mask, a 6 nm-thick Ca layer and a 125 nm-thick Ag layer were deposited via vacuum thermal evaporation (VTE) to form the bilayer cathode.

### 2.3. Thin Film and Device Characterization

The emissive thin films based on blended emitting polymers or the copolymers were characterized in terms of their optical, electronic, and photophysical properties using Spectroscopic Ellipsometry (SE) and Photoluminescence (PL). In addition, in order to examine the possible energy transfer mechanism between the different units of both blends and copolymers, absorbance measurements were conducted for dopants and PL for the hosts. Then, the emissive layers were inserted in the produced OLEDs and were investigated through Electroluminescence (EL) Spectroscopy.

The SE measurements were conducted using a phase-modulated ellipsometer (Horiba Jobin Yvon, UVISEL, Europe Research Center—Palaiseau, France) from the near IR to far UV spectral region 1.5–6.5 eV with a step of 20 meV at a 70° angle of incidence. The SE experimental data were fitted to model-generated data using the Levenberg–Marquardt algorithm taking into consideration all the fitting parameters of the applied model.

The absorbance measurements were conducted using the set-up of Theta Metrisis (model FR UV/VIS) (Theta Metrisis S.A., Athens, Greece). Combining deuterium and halogen light sources, all measurements were performed in the 300–700 nm spectral range.

The PL and EL measurements of the active layers and the final OLED devices, respectively, were measured using the Hamamatsu Absolute PL Quantum Yield measurement system (C9920-02) and the External Quantum Efficiency (EQE) system (C9920-12) (Joko-cho, Higashi-ku, Hamamatsu City, Japan) which measures the luminance and light distribution of the devices. The PL spectra are recorded upon the excitation wavelength of 380 nm.

## 3. Results and Discussion

### 3.1. Spectroscopic Ellipsometry

The Spectroscopic Ellipsometry technique is a well-established characterization tool for determining optical constants. It is a non-destructive method that is free from the limitations associated with other methods that require physical contact with the film. In this study, the optical properties of the emissive materials were examined using the Vis-fUV SE method in the spectral region 1.5–6.5 eV. The Tauc-Lorentz model was used to derive the optical properties of each emitting phase, which was used and incorporated into either the copolymers or blended materials [27,28,29]. In addition, we can also obtain valuable information through the SE analysis, such as the thickness values of emissive films.

**Figure 1:**
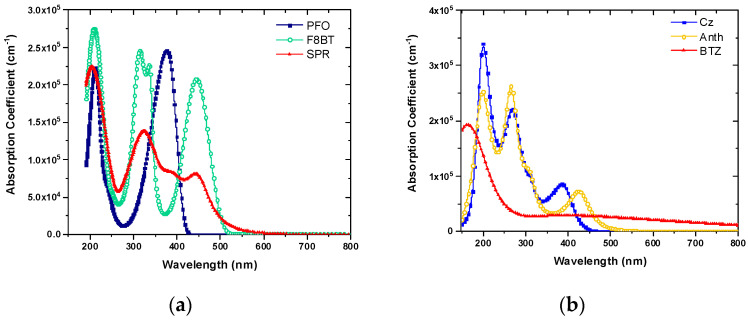
The absorption coefficient of each phase, which was incorporated in the (**a**) two and three blended systems and (**b**) copolymers and terpolymers.

After the fitting process, the absorption coefficient versus wavelength was calculated for each emissive polymer contained in the blended two-phase and three-phase systems and for each monomer–chromophore incorporated in both copolymers and terpolymers, as shown in Figure 1a,b, respectively. Thus, Figure 1a,b depict the resulting spectra of the absorption coefficient used for the calculation of the spectral overlap integral *J*(*λ*), which are presented as follows.

### 3.2. Förster Resonance Energy Transfer (FRET)

Control in the Förster resonance energy transfer mechanism between emitters is regarded as the most important factor for achieving white light emission. It is well known that in the host–guest system, the absorbed energy in fluorescent organic molecules excites electrons from the ground state to a higher excited state, but a part of the energy can be released to lower excited states throughout the internal conversion of the electron before relaxation and light emission. The spectral overlap between the host’s PL emission and the absorbance of the dopants in all studied emitting films is investigated further to find out if the energy transfer mechanism can possibly take place between the host and the dopants.

**Figure 2:**
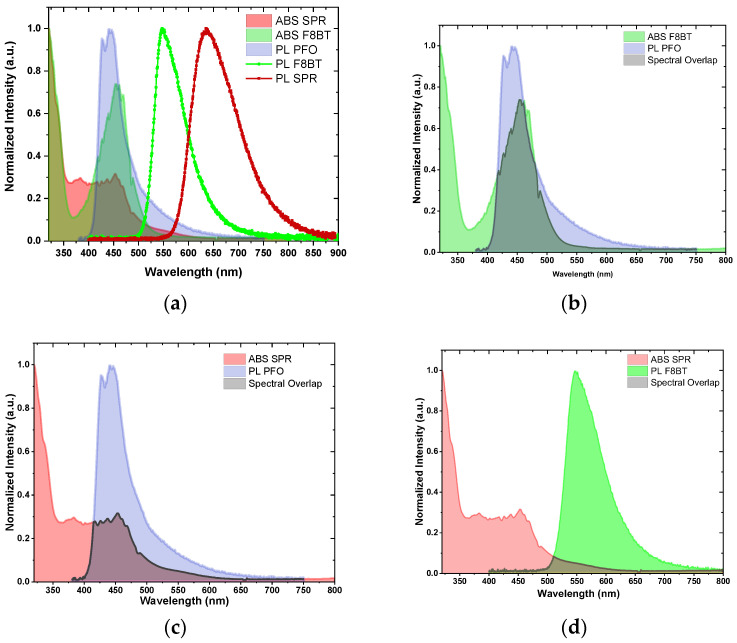
The overall correlation between (**a**) the absorbance of the SPR and F8BT units with the PL emissions of PFO, F8BT, and SPR. The examined spectral overlap between (**b**) PFO and F8ΒΤ, (**c**) PFO and SPR, and (**d**) F8BT and SPR.

Figure 2a displays the absorption and PL emission profiles of the dopants, F8BT and SPR, accompanied by the PL emission of PFO. It is obvious that the absorption spectra of the F8BT and SPR overlap strongly with the emission spectrum of the PFO, whereas a lower spectral overlap is observed in the case of SPR absorption and F8BT PL. The degree of overlap between the absorption and PL emission peaks of the emissive materials is used to predict the energy transfer efficiency. It is possible to quantify the amount of energy transferred between the hosts and dopants by calculating the relative area of the overlap $\;E^{overlap}$ between the residual absorption of each dopant within the wavelength range of each host’s respective PL emission in UV-Vis absorption spectra and the total area of the PL emission of the hosts $E^{emission}$ [29]. Finally, the calculated percentage is given using the following equation:

(1) $$E=\frac{E^{overlap}}{E^{emission}}\times 100\%$$

Figure 2b–d show the spectral overlap between the PL emission of PFO and absorption of F8BT, the PL emission of PFO and absorption of SPR, and the PL emission of F8BT and absorption of SPR, respectively. As shown in Figure 2b, the emission spectrum of PFO overlaps very well with the absorption band of F8BT in the region between 400 and 525 nm, and this is confirmed by the calculation of a 62% percentage. In the case of PFO-SPR, a smaller spectral overlap within the range of 400–600 nm is observed, and the percentage is calculated as 33%. Therefore, the energy-transfer mechanism is feasible, and this fact is significant for both approaches, either two- or three-phase blend polymers. Finally, the frustrated overlap of F8BT-SPR is observed, and it is much lower than that of PFO-SPR and PFO-F8BT, as indicated by the 6% calculated percentage.

**Figure 3:**
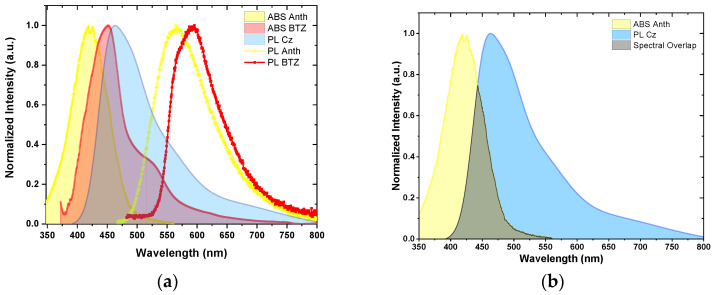

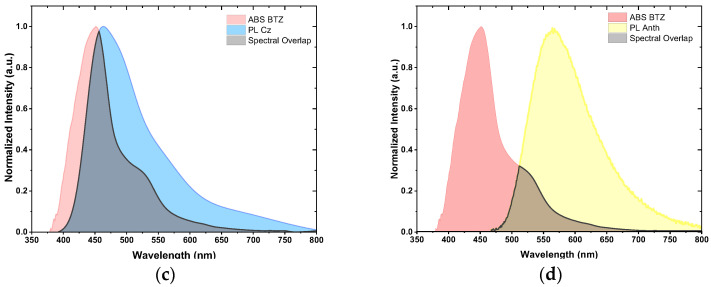
The overall correlation between (**a**) the absorbance of the Anth and SPR units with the PL emissions of Cz, Anth, and BTZ. The examined spectral overlap between (**b**) Cz and Anth, (**c**) Cz and BTZ, and (**d**) Anth and BTZ.

For the synthesized emitting copolymers, we follow the same methodology in order to study if it is possible for the energy transfer between the host and dopants to occur. Figure 3a depicts the absorption and PL spectra of Anthracene (Anth) and Benzothiadiazole (BTZ) together with the PL emission of Carbazole (Cz). It is evident that spectral overlaps are observed between the Cz and Anth and BTZ but also between Anth and BTZ. 

To be clearer, Figure 3b–d show the spectral overlaps between the PL emission of Cz and absorbance of Anth, the PL emission of Cz and absorbance of BTZ, and the PL emission of Anth and absorbance of BTZ, respectively. The overlap between Cz and Anth covers the spectra range 400–550 nm, and the degree is calculated at 24%, indicating sufficient energy transfer from the Cz to the Anthracene. The spectral overlap between Cz and BTZ is significantly broader at the spectral range 400–750 nm. The calculated percentage for this case is equal to 58%, and this confirms that it is possible for more efficient energy transfer to take place. At this point, it is noteworthy that this huge spectral overlap manifests as a large fraction of energy being transferred from the Cz to the BTZ, and this fact is favorable for both cases of the copolymers based on two and three chromophores. The last combination of Anth–BTZ proves the lower spectral overlap within the range 470–750 nm, and this is also verified from the respective percentage value, which is calculated at 19%. According to these results, we assume that the sequential energy transfer happens from the Cz to Anthracene and BTZ, but also that there is a possibility that the energy transfer occurs from the Anthracene to BTZ. All the after mentioned results concerning the calculation of E values for all the possible combinations of host and dopants are written in Table 3.

A second approach is to estimate the overlap degree between the absorption spectrum of the acceptor with the emission spectrum of the donor, by calculating the overlap integral between them. More specifically, the overlap integral, *J*, is the integral of the spectral overlap between the donor emission and the acceptor absorption, which is given by the following:

(2) $$J\left(\lambda \right)\sim \int_{0}^{\infty }\varepsilon_{A}\left(\lambda \right)F_{D}(\lambda )\lambda^{4}d\lambda$$

where $F_{D}(\lambda )$ is the donor emission spectrum normalized to its area, and ε_A_ (λ) is the molar extinction coefficient of the acceptor, measured in M^−1^·cm^−1^ [20,25,30]. The calculated values J(λ) are listed in Table 3. According to the results, the Förster type of energy transfer between the PFO monomers and each monomer of SPR and F8BT, as well as between SPR and F8BT, is confirmed. The calculated values J(λ) of copolymers also validate the Förster energy transfer between the Cz unit and each unit of Anth and BTZ, as well as between Anth and BTZ.

### 3.3. Photoluminescence

**Figure 4:**
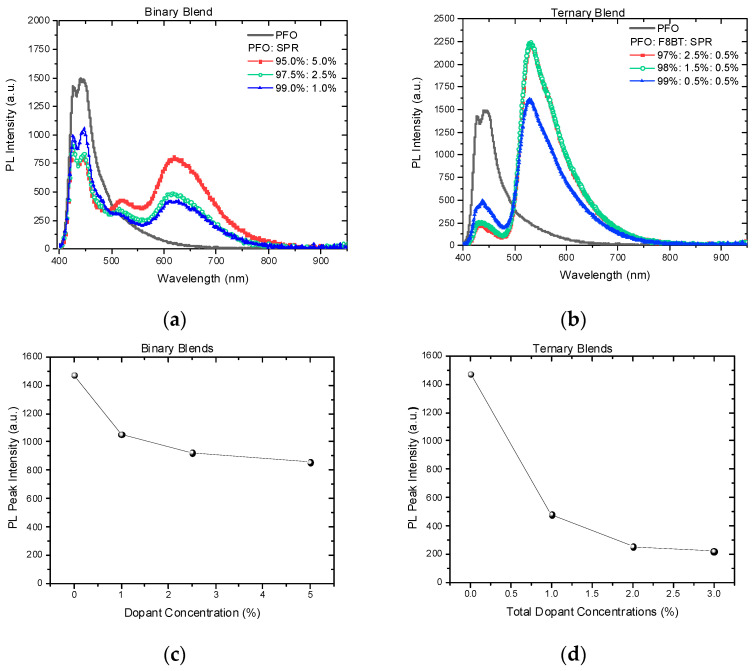
Emission spectra of PFO in the absence and the presence of (**a**) SPR and (**b**) F8BT-SPR and PFO PL peak intensity versus dopant concentration for the blended systems (**c**) PFO-SPR and (**d**) PFO-F8BT-SPR.

Figure 4 shows the emission spectra of the produced (a) binary and (b) ternary blends with different doping concentrations, respectively. In all cases, the characteristic emission of the PFO (400–500 nm) exhibits a strong influence on the doping concentration. In particular, in binary blends (PFO-SPR), a gradual decrease in the PFO emission intensity with the addition of SPR is obtained, and at the same time, the intensity of the SPR unit (550–800 nm) is strengthened. The quenching mechanism is due to the partial energy transfer from PFO to SPR. On the other hand, in ternary blends (PFO-F8BT-SPR), a significant quenching of the PFO fluorescence intensity by increasing the total doping concentration of F8BT-SPR is observed (Figure 4b). This indicates an almost complete energy transfer from PFO to dopants.

Figure 4 also shows the PFO PL peak intensity versus dopant concentration for the respective examined (c) binary and (d) ternary systems. In the case of a binary system, the PL intensity peak decreases gradually as the dopant concentration increases. On the other hand, in the ternary system, the PL peak intensity is reduced significantly, when the total doping concentration of F8BT-SPR is increased. This fact may be attributed to the energy transfer mechanism that quenches the fluorescence intensity of the PFO and reduces its excited-state lifetime while increasing the emission intensity of the acceptors F8BT-SPR [20,25,30,31].

**Figure 5:**
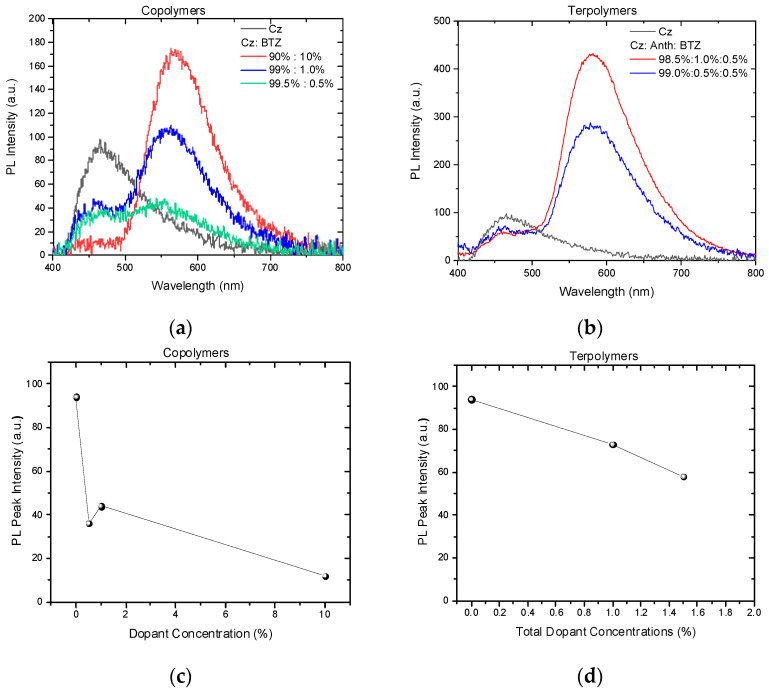
Emission spectra of Cz in the absence and the presence of (**a**) BTZ, and (**b**) Anth-BTZ and Cz PL peak intensity versus the dopant concentration for the copolymers (**c**) Cz-BTZ and (**d**) Cz-Anth-BTZ.

The same analysis was conducted for copolymer and terpolymer emissive layers. Figure 5 shows the emission spectra of the produced (a) copolymers and (b) terpolymers in different concentrations, respectively. Firstly, the general result is that the maximum emission intensities of the copolymers (Cz-BTZ) decrease with a decreasing acceptor concentration. The possible reason for this phenomenon is radiative migration due to self-absorption. When the concentration of BTZ reaches 10% *w*/*v*, the emission of Cz is almost completely quenched and the emission of BTZ is enhanced and is the dominant one. The damping of Cz is attributed to the complete energy transfer from Cz to the BTZ unit. Similarly, in the case of terpolymers (Cz-Anth-BTZ), the emission intensity of Cz decreases in the presence of Anth-BTZ. Increasing the total concentration of Anth-BTZ results in the decrease in the PL intensity of Cz and in the enhancement of the combined PL intensity of Anth-BTZ.

Further analysis, regarding the correlation of the Cz maximum PL intensity values with the dopant concentration, is illustrated in Figure 5 for the (c) copolymers and (d) terpolymers. In both cases, it is obvious that the intensity of the PL peak originating from the Cz emission decreases significantly with an increasing doping concentration. Thus, relying on these results, it is confirmed that the reduction in the PL intensity peak, which is assigned to the host unit, occurs due to the efficient energy transfer between the host and the dopants.

**Figure 6:**
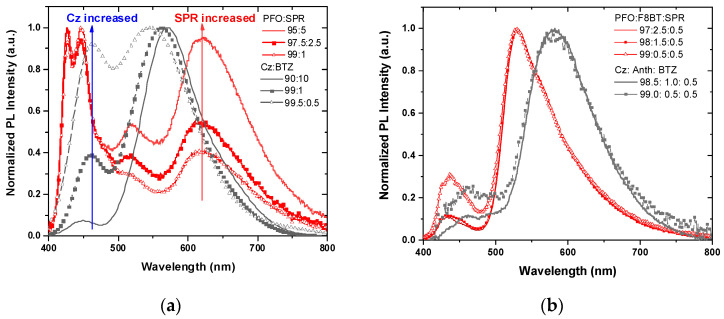
Comparison of normalized PL spectra between the (**a**) binary PFO-SPR with Cz-BTZ copolymers and (**b**) ternary PFO-F8BT-SPR with terpolymers Cz-Anth-BTZ.

The potential use of the studied films in achieving broad emission in the visible spectral region is determined by the controllable FRET mechanisms between the host and dopants. The emission tuning based on two different emitters, the blue and red color, enables the definition of white light emission. Figure 6a shows the normalized PL emission spectra of the binary blended polymers in comparison with the PL emission of copolymers. It is obvious that the emission profile of both binary blended polymers and copolymers covers the whole visible range from 400 to 800 nm. We further analyzed the PL spectra using the deconvolution method using the required number of Gauss oscillators. The extracted results for *λ*^max^ and full width half maximum (FWHM) of each oscillator are summarized in Table 4. The respective thicknesses of the studied films, derived by SE analysis, are also included in Table 4.

According to our results, in the binary blended polymers PFO-SPR, the main emission peak attributed to the PFO unit is observed, and a weaker emission peak can be also detected, which is assigned to SPR. Here, it is notable that the characteristic emission intensity (*λ*^max^ = 615 nm) of SPR was gradually enhanced by increasing the SPR concentration. The highest doping concentration of SPR led to two characteristic emission peaks covering the entire visible range, but in the region between 490 and 570 nm, the intensity peak remains low. The PL peak (*λ*^max^ = 510 nm) can be attributed to the PFO defects, as it is well established that polyfluorene-based materials often exhibit a long-range emission around 2.2–2.3 eV [32]. On the contrary, in the copolymers Cz-BTZ, the dominant PL peak is located at approximately 560 nm, which is associated with the red chromophore BTZ. This means that in copolymers Cz-BTZ, the energy transfer mechanism plays a significant role in the PL emission profile, as the lower intensity peak comes from the blue chromophore. It is also remarkable to refer that in the case of the copolymer with the lowest concentration of BTZ, two main peaks are distinguished to span the entire visible range. The emission intensities of the blue and red chromophores are evenly balanced. This suggests that the energy-transfer mechanism between the two chromophores is optimized, which efficiently contributes to the mixing of blue and orange–red emission. Therefore, it is evident that the PL spectra can be tuned based on the ratios both in the binary blends and copolymers.

Figure 6b displays the normalized PL spectra of the ternary blended polymers (PFO-F8BT-SPR) and the terpolymers bearing three chromophores (Cz-Anth-BTZ), to facilitate a comparison. One can see that the PL emission spectra based on three emitters present a dominant peak, which originates from the dopant’s units. This fact can be interpreted based on the assumption that the final intensity of the emission spectrum is a sum of the total energy transfer from the host to the dopants. More specifically, for the ternary blended polymers, the PL intensity of PFO is lower compared to the other two emitters F8BT and SPR due to the efficient energy transfer. In the case of the highest concentration of PFO in ternary blends, the contribution from the PFO emission was much more evident. However, the PFO intensity is weak, so there is no balanced tuning between the emitters. For the other approach based on terpolymers, the PL profiles reveal that the main peak appearing in all cases is attributed to the dopant emission. The emission intensity of Cz is attenuated when the doping concentration is increased. Thus, the quenching of the Cz emission can be supported by the efficient energy transfer mechanism from Cz to Anthracene and BTZ.

Note that in the case of both the binary blend and copolymer, the dopant concentration significantly affected the peak intensities in the PL spectra, which were attributed to the two emitters (blue-red). More specifically, in the case of the binary, the different doping concentrations influence the PL intensity peak of the red emitter, whereas, in copolymers, the different doping concentrations influence the PL intensity peak of the blue emitter. Therefore, efficient energy transfer from the wide-gap homopolymer to the narrow band-gap unit may happen due to a strong interchain interaction. This difference between the two emitting systems may be assigned to the rate of energy transfer. This rate is highly dependent on many factors, such as the spectral overlap, the relative orientation of the transition dipoles, and, most importantly, the distance between the donor and acceptor molecules. Regarding the three emitters, the different doping concentrations did not effectively contribute to the tuning of the emission of each emitter to span the entire visible range. Thus, adjusting the doping concentration in two emitters leads to easier tuning and results in a balanced two-color emission compared to three emitters.

As a means of elucidating which combination of emitting systems will generate white light, CIE (Commission Internationale de l’Éclairage) coordinates were calculated through the respective PL emission spectra. The CIE color system is the most widely used colorimetric standard so far. The CIE-1931 coordinates describe how the human eye perceives the color emission from any light source using Cartesian coordinates. The CIE specifies that the coordinates for the point of white light are (0.33, 0.33) [33,34].

**Figure 7:**
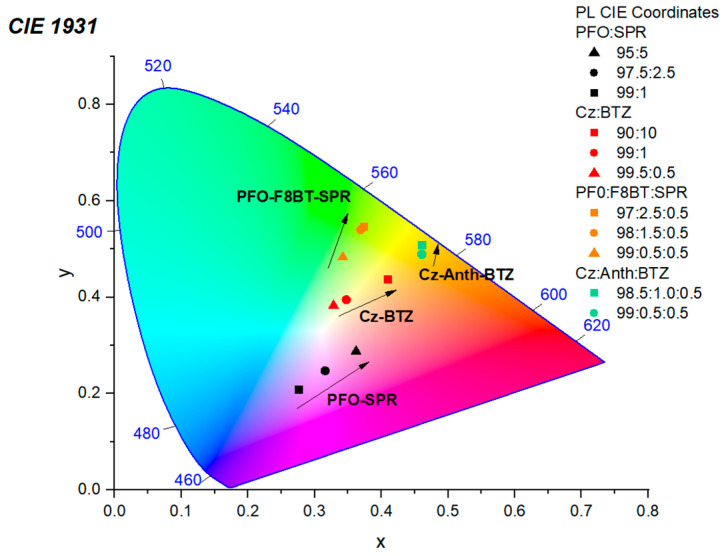
CIE diagram of PL emissions of the studied materials. The arrows denote the increase in the percentage of the dopant.

Figure 7 shows the CIE diagram of coordinates corresponding to the PL spectra. As can be seen, by increasing concentrations of the red-emitting polymer SPR (denoted by arrows) in the binary blends, the CIE coordinates tend to approach the white region. On the other hand, the opposite occurs in the case of copolymers Cz-BTZ, in which by reducing the concentration of the red dopant, BTZ (the arrow denotes the increase of BTZ), the CIE coordinates approach the ideal white light emission. Furthermore, concerning the CIE coordinates of both ternary blends and terpolymers, they are quite far from the white light emission. Here, it is also observed that in both cases, the reduced doping concentration shifts the coordinates toward the white region.

### 3.4. Electroluminescence

**Figure 8:**
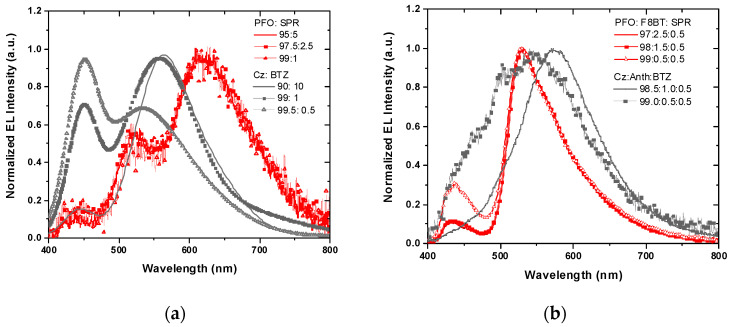
Comparison of normalized EL spectra between (**a**) binary PFO-SPR with Cz-BTZ copolymers and (**b**) ternary PFO-F8BT-SPR with terpolymers Cz-Anth-BTZ.

The blended and synthesized polymers were applied as emitting layers in single-layer OLEDs, having the device structure of ITO/PEDOT:PSS/emitting layer/Ca/Ag. The electro-optical properties and the performance of the fabricated devices were investigated. Figure 8 shows the comparison of the respective normalized EL spectra between the (a) binary blended system and copolymers and (b) ternary blended system and terpolymers. Concerning the analysis of EL spectra using the deconvolution method, the calculated values of *λ*^max^ and FWHM are summarized in Table 5. It is obvious that for all studied cases, the EL emission spectra covered the spectral region from 400 to 800 nm.

**Figure 9:**
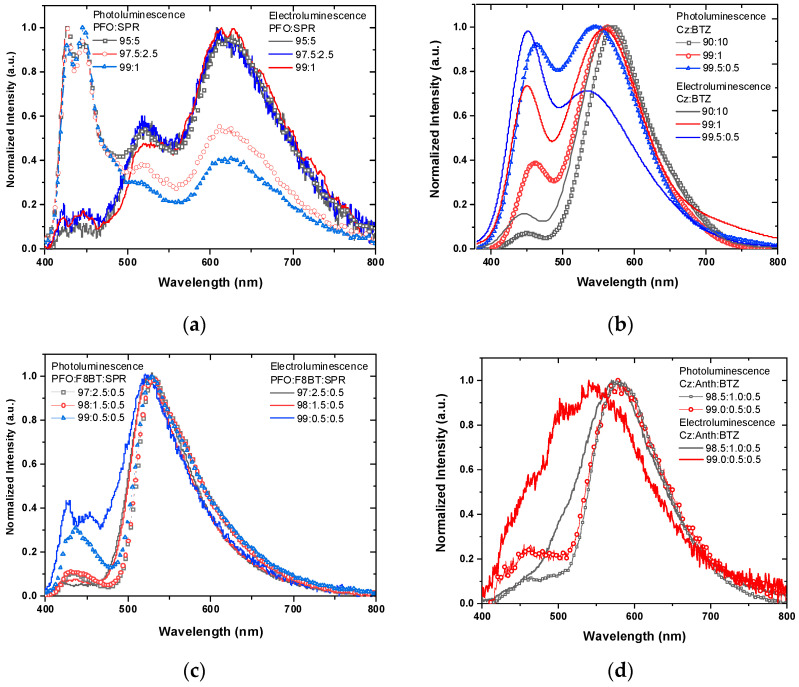
Normalized EL spectra (lines) of OLEDs based on the emitting layers (**a**) binary, (**b**) copolymer, (**c**) ternary, and (**d**) terpolymer, respectively; for a better evaluation of the EL characteristics, the corresponding PL spectra are also incorporated (lines with symbols).

For a better evaluation of the EL characteristics, Figure 9 shows, comparatively, the EL (lines) and PL (lines with symbol) spectra of emitting layers based on the (a) binary, (b) copolymers, (c) ternary, and (d) terpolymers, respectively. In all studied emitting layers, a blue shift of the EL spectra is observed, when comparing to that of PL. If FRET was the only mechanism controlling the emission of WOLED devices, the relative intensities of host and dopant emissions would be equivalent in the corresponding PL and EL spectra. However, this is not valid in all cases examined in our studies, and the relative intensities are substantially different between the PL and EL spectra. In the case of PL emission, it has been proven that the energy-transfer process plays a significant role. Particularly, for all cases, energy transfer can possibly occur, as found based on the evaluation of the spectral overlap between the PL emission of each host and the absorbance of each dopant. On the other hand, it is well established that the EL emission depends not only on the energy transfer mechanism but also on the charge trapping.

As depicted in Figure 9a, in the case of binary PFO-SPR, a comparison between the PL and EL profiles clearly shows substantial differences. The EL emission profiles of all binary blends present a dominant peak at approximately 610 nm, which is attributed to the SPR moiety, and this peak is independent of the doping concentration. Furthermore, the emission of PFO is completely quenched compared to the PL emission profile. Therefore, we can assume that the EL emission originated from other exciton species than those responsible for the PL. This fact can account for the charge trapping in the SPR moiety, apart from energy transfer from the PFO to SPR. In Figure 9b, one can see that significant differences between the PL and EL spectra are also observed in the studied copolymers Cz-BTZ. More specifically, in all copolymers, the EL emission spectra exhibit the main peak at 560 nm, which is associated with the BTZ moiety. It is also important to mention that the relative intensity of Cz is reinforced strongly compared to the PL emission when the doping concentration of BTZ decreased. These EL spectra reveal that the dual emission is a result of the combination of the energy-transfer mechanism and the charge trapping in the Cz unit. It was found that when the concentration of BTZ is generally low (e.g., <1%) apparent dual emissions are displayed at 450 and 560 nm, respectively, which are strong enough to render color balance between blue and red–orange emission to achieve white light.

In all ternary blends (Figure 9c), the tendency of the EL emission band presents similarities with the PL. However, upon decreasing the doping concentrations of both F8BT and SPR, the relative intensity of the PFO peak is more pronounced in the EL spectrum compared to the PL spectrum, and this means that the charge trapping into the PFO unit is more efficient. Finally, the EL emission of terpolymers (Figure 9d) is broader and featureless compared to that of PL. The increase in the Cz concentration enhances the broadening, as well as the blue-shifting, of the emission.

In conclusion, the EL emission of the host is decreased, mainly for the cases of binary and ternary blends, which may be due to the cascade mechanism for the charge injection that favors the exciton formation in the emitter with the lower band gap. On the contrary, in the EL emission profile of copolymers and the ternary blend with the ratio 99:0.5:0.5, the intensity of the host is enhanced, and here, the possible reason is the partial energy transfer from the host to the dopants due to the charge trapping in the host. Finally, in the terpolymers, a featureless and broad EL emission is derived covering the visible spectral range.

**Figure 10:**
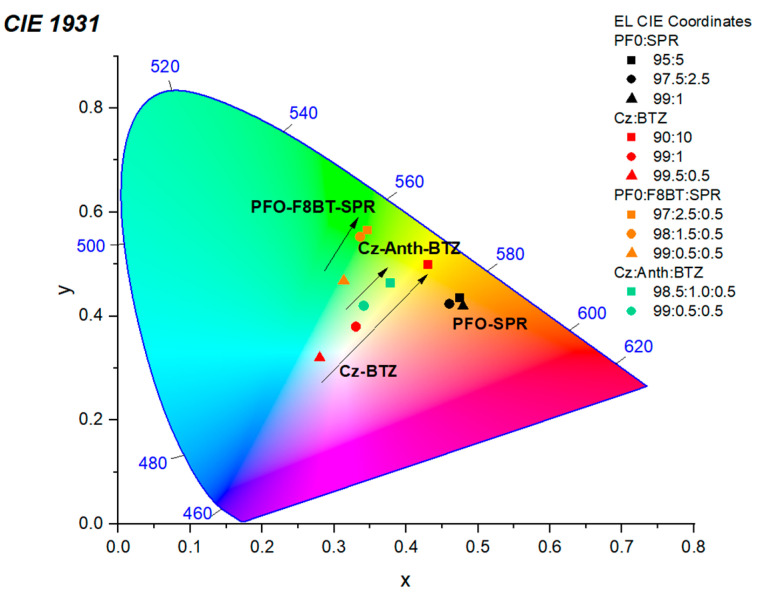
CIE diagram of coordinates derived from EL emissions of the fabricated WOLEDs. The arrows denote the increase in the percentage of the dopant.

Another important feature to evaluate is the color characteristics, including CIE color coordinates (x, y), of the fabricated OLEDs. To better explore this aspect, Figure 10 depicts the CIE coordinates of the studied materials derived from the EL spectra. The CIE coordinates of binary blends is measured representatively at (0.46, 0.42), which are not close to the white region due to the stronger emission of SPR in relation to PFO. On the other hand, the CIE coordinates of copolymers tend to be the values of the ideal white emission (0.33, 0.33). By adjusting the ratio between the two units, Cz and BTZ, and with a dopant ratio <1%, a balanced blue and red emission achieved, and the CIE coordinates are located very close to the pure white-light point with values (0.28, 0.31). These values indicate that this emitting system can be used to generate white-light emission that is well-suited for lighting applications.

As can be seen, the CIE coordinates of ternary blends approach the white region, by increasing the relative ratio of PFO. The perceived emission color of the mixed emitters can be adjusted from green to nearly white, and the obtained CIE coordinates are calculated (0.31, 0.47). Concerning the CIE coordinates of terpolymers, it has been observed that elevating the concentration of Cz leads to the white region being approached, and the obtained coordinates values are equal to (0.34, 0.42).

### 3.5. Performance of OLED Devices

**Figure 11:**
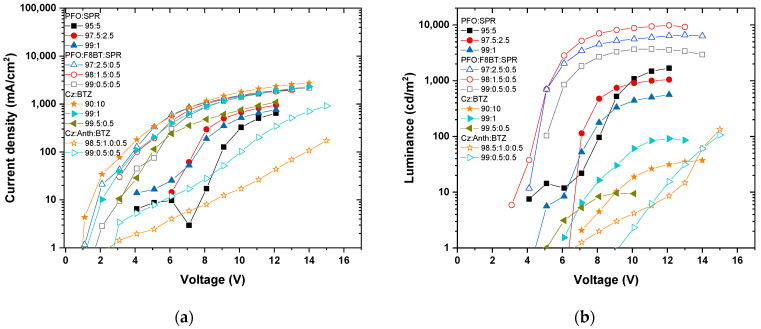
The logarithmic plot of characteristic curves of (**a**) Current density–Voltage (J–V) and (**b**) Luminance–Voltage of the fabricated OLED devices.

Figure 11 shows the logarithmic plot of (a) Current density–Voltage (J–V) characteristic curves and (b) Luminance–Voltage of the fabricated WOLEDs in order to provide additional insights for the operational-electrical characteristics of the devices. The blended materials exhibit higher Luminance values compared to the novel emitting copolymers and terpolymers. Generally, further investigation is needed to enhance the functionalization of produced WOLED devices in order to attain higher efficiency. At this point, it is also remarkable to see that the materials based on three emitters present better luminance performance than the two.

For lighting applications, the color quality and stability must also be considered towards the performance of OLEDs. Detailed characteristics of produced devices, such as CIE color coordinates (x, y), color rendering indexes (CRI), and correlated color temperatures (CCT) [35,36,37], are listed in Table 5. Among all the studied cases, the most encouraging results for the CRI values are obtained from the binary blends and copolymers.

**Figure 12:**
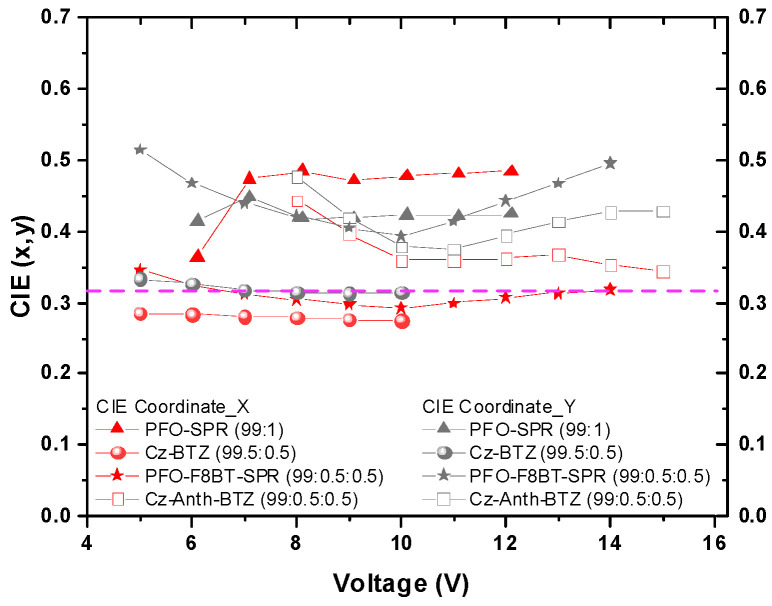
The evolution of EL Chromaticity Coordinates for the WOLED devices, which showed the closest coordinates to the ideal white point at an increasing applied voltage (The dashed line represents the CIE Coordinates of the ideal white point (0.33, 0.33).

The effect of increasing the applied voltage on the shift in the color of the emitted light from WOLED is also examined. This is a crucial factor since the increment in voltage usually results in an increase in emitted light intensity, which may end up causing the unbalanced mixing of emitters. Figure 12 shows the evolution of the EL CIE coordinates only for the WOLED devices, which showed the closest coordinates to the ideal white point, for each examined case. Specifically, the coordinates were measured during device operation and recorded after the turn-on voltage. One can see that the CIE coordinates of copolymer Cz-BTZ demonstrate superior color stability with no variations since the voltage is increased. These materials are promising candidates for lighting applications, as the CIE coordinates are very close to the ideal white point and the CRI value reaches 82. It is a great achievement for WOLED technology, as it is well known that the CRI value of binary-emitter WOLEDs can be boosted from the 63 to 80 threshold [38].

## 4. Conclusions

In conclusion, we have presented two distinct approaches that rely on light-emitting blended polymers or copolymers to realize pure white light emission. Two potential options were explored for blended systems. The first approach was based on mixing two emitters, a red, SPR, with a blue one, PFO. The second approach involved blending three emitters, red SPR with green F8BT and blue PFO. In all cases, PFO was dominated in the percentage and acted as a host. In the case of novel copolymers, the blue Cz derivative was used as the host, which was incorporated with the red chromophore BTZ, and for the terpolymers, the yellow Anth chromophore was added to them. In all doping systems, different ratios of the constituents were examined to tune the emissions and realize white light emission.

At first, for all emitting systems, it was verified that the energy transfer was feasible between the host and the dopants, highlighting the possibility of the management of the emission of each constituent to attain the coverage of the entire visible range. Following this, the emitting polymers were applied as emitting layers in single-layer WOLEDs. Comparing PL and EL emission revealed that charge trapping had a significant impact on EL emission, in addition to the energy transfer mechanism. Mainly, in the case of the Cz-BTZ copolymer, incomplete energy transfer from the blue Cz backbone to the orange-red BTZ moiety resulted in two significant emissions with equal intensity when the doping concentration of BTZ is less than 1%. Specifically, controlling the device’s emission color through a balance of emission between the two units, Cz and BTZ, was achieved, thereby leading to a broad white light emission with satisfying CIE coordinates of (0.28,0.31). Moreover, the color coordinates of the resulting white-light emission remained extremely stable over a wide range of increasing driving voltages. Also noteworthy is that the CRI value obtained for the WOLED was 80, which makes it highly promising for lighting applications. Thus, compared to doping based on blended polymers, chemical synthesis was more efficient, which was conducive to achieving white light emission, as the emission tuning of chromophores was easier by controlling the doping concentration. Overall, the solution-processable WOLED based on copolymers offers an innovative pathway for high-quality emission.

## Figures and Tables

**Table 1:** Blend weight ratios and code-names.

Blend Type	Blend Code					
	Binary#1	Binary#2	Binary#3	Ternary#1	Ternary#2	Ternary#3
PFO:SPR	95:5	97.5:2.5	99:1			
PFO:F8BT:SPR				97:2.5:0.5	98:1.5:0.5	99:0.5:0.5

**Table 2:** The co-monomer ratios of the copolymers and code-names.

Copolymer Type	Copolymer Code				
	Copolymer#1	Copolymer#2	Copolymer#3	Terpolymer#1	Terpolymer#2
Cz:BTZ	90:10	99:1	99.5:0.5		
Cz:Anth:BTZ				98.5:1.0:0.5	99:0.5:0.5

**Table 3:** Calculated values of the parameter E and overlap integral J(λ).

Material	E (%)	J(λ) (M^−1^·cm^−1^·nm^4^)
PFO-F8BT	62	$2.164\;\times \;10^{10}$
PFO-SPR	33	9.451 $\times {\;10}^{9}$
F8BT-SPR	6	$6.176\;\times \;10^{8}$
Cz-Anth	24	2.001 ${\times \;10}^{10}$
Cz-BTZ	58	1.426 $\times \;10^{12}$
Anth-BTZ	19	2.017 $\times \;10^{12}$

**Table 4:** The obtained thickness values, based on the SE analysis, parameters of the deconvolution analysis of PL spectra, and the values of PL CIE coordinates.

Material	Ratio (%)	Thickness (nm)	$\lambda_{PL}^{max}$ (nm)	FWHM (nm)	CIE Coordinates	
					x	y
PFO-SPR	95:5	48.3	424, 444, 510, 615, 651	12, 30, 70, 59, 119	0.3621	0.2877
	97.5:2.5	42.8	424, 444, 510, 615, 651	11, 28, 81, 49, 118	0.3156	0.2473
	99:1	40.4	424, 444, 510, 615, 651	12, 25, 74, 22, 131	0.2762	0.209
Cz-BTZ	90:10	21.0	445, 567, 634	35, 68, 113	0.4097	0.4378
	99:1	32.0	454, 560, 638	41, 84, 168	0.3473	0.3946
	99.5:0.5	9.9	459, 537, 616	51, 83, 89	0.3282	0.3826
PFO-F8BT-SPR	97:2.5:0.5	42.6	438, 525, 555, 602	30, 29, 54, 111	0.3737	0.5471
	98:1.5:0.5	43.5	439, 524, 554, 602	32, 29, 55, 112	0.3688	0.54
	99:0.5:0.5	40.4	439, 523, 551, 586	33, 28, 54, 129	0.3422	0.4832
Cz-Anth-BTZ	98.5:1.0:0.5	53.7	467, 570, 619	43, 66, 114	0.4607	0.5081
	99:0.5:0.5	60.5	467, 574, 622	44, 66, 110	0.4607	0.4888

**Table 5:** The obtained parameters of deconvolution analysis of EL spectra and electrical-operational characteristics of the produced OLED.

Material	Ratio (%)	$\lambda_{EL}^{max}$ (nm)	FWHM (nm)	EL CIE Coordinares (x, y)	Luminance (cd/m^2^)	CRI	CCT (K)	* V_on_ (V)
PFO-SPR	95:5	438,517,613,642	36,47,61,142	(0.47, 0.44)	1033	94	2515	4.1
	97.5:2.5	442, 517,612,651	43,44,72,145	(0.46, 0.42)	1038	94	2663	6.5
	99:1	441,519,613,654	43,41,73,150	(0.48, 0.42)	561	95	2620	4.5
Cz-BTZ	90:10	443,555,613	60,64,104	(0.43, 0.50)	37	60	4063	7.1
	99:1	448,551,614	41,91,138	(0.33, 0.38)	91	70	5498	6.1
	99.5:0.5	447, 520,580	17,40,75	(0.28, 0.31)	10	82	8000	5.1
PFO-F8BT-SPR	97:2.5:0.5	421,440,526,572	14,16,47,94	(0.34, 0.56)	7474	44	5264	4
	98:1.5:0.5	423,441,525,573	13,17,47,98	(0.34, 0.56)	9838	49	5697	3.1
	99:0.5:0.5	424,448,522,562	16,27,56,168	(0.31, 0.47)	3321	74	7698	5.1
Cz-Anth-BTZ	98.5:1.0:0.5	481,569,614	60,83,142	(0.44, 0.48)	107	60	2787	7
	99:0.5:0.5	477,561,607	56,115,132	(0.34, 0.42)	134	74	2974	9

* V_on_: Turn-on Voltage at 1 cd/m^2^

## Data Availability

Data are contained within the article.
